# Superior accuracy of external ventricular drain placement using electromagnetic navigation compared to freehand insertion: A retrospective cohort study

**DOI:** 10.1016/j.bas.2025.104313

**Published:** 2025-07-02

**Authors:** Silas H. Nielsen, Kasper A. Henriksen, Rune Rasmussen

**Affiliations:** Department of Neurosurgery, Copenhagen University Hospital, Copenhagen, Denmark

**Keywords:** External ventricular drainage, Electromagnetic navigation, Kakarla grading, Ventriculostomy, Neurosurgical training

## Abstract

• EM-guidance significantly enhances the accuracy of EVD placements compared to freehand insertion.
• EM-guidance was more frequently utilized in anatomically challenging cases characterized by significantly smaller ventricular systems and a higher frequency of midline shift.
• EM-guidance was primarily used by less experienced neurosurgeons in training.
• EM-guidance is recommended for routine neurosurgical practice, particularly in anatomically challenging cases.

## 1 Introduction

The placement of an external ventricular drain (EVD) is among the most common neurosurgical procedures, frequently performed by neurosurgical trainees or residents. An EVD serves two main purposes: monitoring and treating elevated intracranial pressure and providing acute, life-saving pressure relief by draining cerebrospinal fluid (CSF) into an external compartment for patients with severely elevated ICP due to intracranial hemorrhage, infection, tumors, or other pathologies.

An EVD is traditionally inserted using the freehand technique guided by anatomical landmarks, typically Kocher's point (Amoo et al., 2021). A recent meta-analysis reports a first-pass success rate of 78 % and an optimal final placement rate of 72 % (Nawabi et al., 2023). Optimal placement is defined as positioning the catheter in the ipsilateral frontal horn or third ventricle, according to the Kakarla grading system. Inaccurate placement can lead to severe complications, including intracerebral hemorrhage and suboptimal cerebrospinal fluid drainage, and the need for catheter repositioning (Toma et al., 2009), underscoring the importance of optimizing this procedure. Although previous studies and systematic reviews have demonstrated that guidance improves placement accuracy compared to the freehand technique, (Nawabi et al., 2023), (Stuart et al., 2021), (AlAzri et al., 2017), (Shtaya et al., 2018), (B. Fisher m.fl, 2021) its adoption as standard practice remains limited. Moreover, existing literature primarily focuses on a single EM-navigation system and in selected patient cohorts (AlAzri et al., 2017), (Mahan et al., 2013).

In this retrospective study, we present a comparative analysis of 249 patients who underwent either freehand (n = 163) or electromagnetically (EM)-guided (n = 86) EVD placement. This study is the first and largest systematic evaluation of the Brainlab EM-navigation system implemented routinely, reflecting typical neurosurgical practice rather than selected cohorts (AlAzri et al., 2017).

The primary aim of this study was to evaluate the accuracy, complication rates, and clinical utility of EM-guided EVD placement compared to traditional freehand techniques in everyday neurosurgical practice.

## 2 Materials and methods

### 2.1 Inclusion

The study was approved by the National Danish Research Ethics Committee (Project Identification: 100249) and conducted at the Department of Neurosurgery, Copenhagen University Hospital. We retrospectively identified all patients who received an EVD between January 1, 2022, and September 30, 2023. This 21-month interval was selected to include the maximum number of cases following the implementation of EM guidance at our center while adhering to the ethics committee's requirement for a purely retrospective design. Patients were identified using the ICD-10 procedure codes: Hydrocephalus external drainage (DC793A) and External drainage of liquor (DI606). Inclusion criteria required preoperative and postoperative neuroimaging (computed tomography [CT] or magnetic resonance imaging [MRI]). Exclusion criteria included reuse of burr holes and newborn patients.

### 2.2 Data collection

Preoperative radiological data included the Evans Index, and the presence of a midline shift as reported on the radiological reports. Postoperative EVD placement accuracy assessment was based on postoperative imaging and independently verified by two authors involved in the study. Accuracy was classified according to the widely used Kakarla Grading System (Kakarla et al., 2008) and subsequently dichotomized as grade 1 (optimal placement in the ipsilateral frontal horn or third ventricle) or non-grade 1 (grades 2–3, representing suboptimal placement in non-eloquent tissue or eloquent tissue, respectively). Electronic Health Records (EHRs) were retrospectively reviewed for treatment indication, EVD placement method (freehand or EM-navigation), surgical side (left or right), number of EVD placement attempts (1 or more; if no number was recorded, one attempt was assumed), surgical experience in years, surgical title (e.g., resident or consultant), supervision, reoperation due to misplacement, postoperative ventriculitis with verified microbial cultures, and treatment-related hemorrhages.

### 2.3 Ventricular drain placement technique

Local anesthesia is administered at the entry site at Kocher's point, followed by a precise stab incision in the skin. A twist drill is then used to create a burr hole, through which the dura is perforated using a perforator. Next, a Silverline® ventricular catheter (8F, Spiegelberg) equipped with a cranial bolt is inserted into the burr hole. The correct placement of the catheter is confirmed by the appearance of cerebrospinal fluid. Finally, the cranial bolt is tightly screwed into the burr hole to secure the catheter.

### 2.4 Freehand technique

Kocher's point is identified using anatomical landmarks: 2–3 cm lateral from the midline at the mid-pupillary line and 1–2 cm anterior to the coronal suture, approximately 11 cm from the glabella. Once identified, the ventricular catheter is inserted perpendicular to the skull, using the external auditory canal and the ipsilateral epicanthus medialis as reference points for alignment.

### 2.5 Electromagnetic guidance

The Kick Electromagnetic Navigation System Cranial from Brainlab and the EM stylet were used for placement. Surgeons performing these procedures underwent specialized training provided by the senior author to ensure proficiency with the system. The navigation system assisted in determining the entry point, direction, and depth.

### 2.6 Setting of procedures

Most procedures in both groups were performed in the operating room. Of the freehand procedures, 12 out of 163 (7.4 %) were performed bedside, while all EM-guided procedures were performed in the operating room.

### 2.7 Statistics

Descriptive statistical measures were computed for each variable. Frequencies and ratios were used for categorical variables, while continuous data were presented as means with standard deviations for normally distributed data, or medians with interquartile ranges for non-normally distributed data. Data distributions were visually assessed.

Categorical variables were compared using the Chi-squared test or Fisher's exact test, depending on sample size. Continuous variables were compared using an unpaired *t*-test for normally distributed data or the Wilcoxon rank-sum test for non-normally distributed data.

All statistical analyses were performed using R software (version 4.3.2; R Foundation for Statistical Computing, Vienna, Austria). P-values below 0.05 were considered statistically significant.

## 3 Results

### 3.1 Cohort demographics

**Fig. 1:**
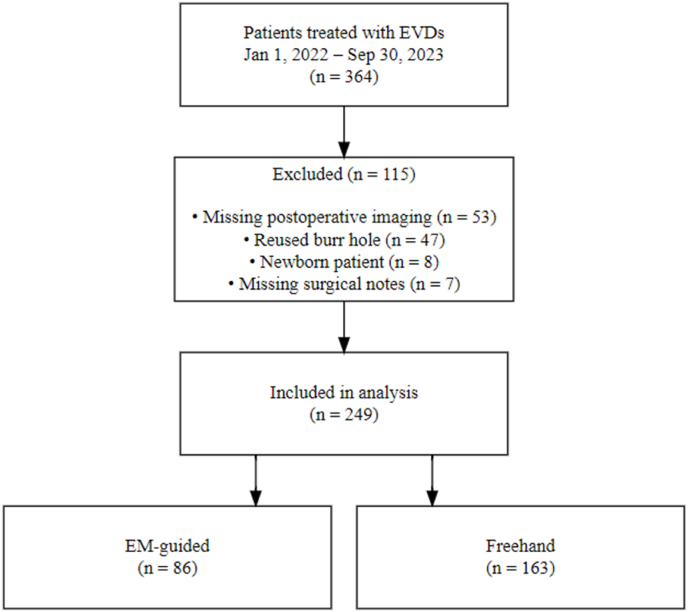
Case inclusion flowchart.

All patients (n = 364) treated with EVDs between January 1st, 2022, and September 30th, 2023, were retrospectively included. The primary reasons for exclusion were missing postoperative imaging (n = 53), reused burr holes (n = 47), newborn patients (n = 8), and missing surgical notes in the electronic health records (n = 7), resulting in a total of 249 included EVDs (Fig. 1).

The majority of patients were female (n = 139, 55.8 %), and the median age was 57.0 years. Placement was performed using EM-guidance in 86 patients (34.5 %), while the remaining 163 patients (65.5 %) underwent EVD placement using the freehand (FH) insertion technique.

No statistically significant difference was observed between the EM and FH groups regarding biological sex. However, the EM cohort was significantly younger than the FH group (Median age EM: 52 years, IQR [22.5] vs. Median age FH: 60 years, IQR [16.5]; p < 0.001), as shown in Table 1.

**Table 1:** Cohort demographics.

Characteristic	ECN = 249^a^	EMN = 86^a^	FHN = 163^a^	p-value
Sex				0.11^b^
Female	139 (55.8 %)	42 (48.8 %)	97 (59.5 %)	
Male	110 (44.2 %)	44 (51.2 %)	66 (40.5 %)	
Age at time of operation				<0.001^c^
Median, (IQR)	57.0, (22.0)	52.0, (22.5)	60.0, (16.5)	

Characteristics of the entire cohort (EC, n = 249), patients with EM-navigation (EM, N = 861), and those with freehand (FH, n = 163) EVD placement. Analyzed variables include gender distribution and median age at the time of operation. Statistical significance was assessed using Pearson's Chi-squared test for categorical data and the Wilcoxon rank sum test for continuous data, indicating a significant age difference (p < 0.0013) but no significant difference in sex distribution (p = 0.112). IQR: Interquartile Range.

a n (%).

b Pearson's Chi-squared test.

c Wilcoxon rank sum test. IQR: Interquartile range, EC: Entire cohort, EM: Electromagnetic-navigation, FH: Freehand.

### 3.2 Placement accuracy and complications

The overall accuracy of EVD placements was 87.1 %. Using electromagnetic guidance for EVD placement significantly improved accuracy compared to FH insertion. EM-guided EVDs had a 7 % higher accuracy rate, with 93.0 % achieving Kakarla grade 1, compared to 84.0 % in the FH group. This difference was statistically significant (Chi-squared, p = 0.044) (Table 2).

**Table 2:** Placement accuracy.

Characteristic	Overall N = 249^a^	EMN = 86^a^	FHN = 163^a^	p-value^b^
Kakarla grading scale				**0.044**
Kakarla 1^c^	217 (87.1 %)	80 (93.0 %)	137 (84.0 %)	
Kakarla 2^d^	25 (10.0 %)	5 (5.81 %)	20 (12.3 %)	
Kakarla 3^e^	7 (2.81 %)	1 (1.16 %)	6 (3.68 %)	

Comparison of placement accuracy across the entire cohort (N = 249), EM-guided (N = 86), and freehand (N = 163) groups, based on the Kakarla grading scale. The table shows percentages of placements achieving Kakarla grade 1 (optimal placement) and grades 2–3 (suboptimal placement), with a statistically significant better outcome in the EM-guided group (p = 0.044). Statistical analysis was conducted using Pearson's Chi-squared test.

a n (%).

b Pearson's Chi-squared test (Kakarla grade 1 vs. grade 2–3) EM: Electromagnetic-navigation, FH: Freehand.

c Karkala 1: Optimal or adequate placement in ipsilateral frontal horn, including tip of the third ventricle

d Kakarla 2: Supoptimal placement in noneloquent tisse; contralateral frontalhorn or lateral ventricle/corpus callosum/interhemispheric fissure

e Kakarla 3: Suboptimal placement in eloquent tissue; brainstem/cerebellum/internal capsule/basal ganglia/thalamus/occipital cortex/basal cisterns

Complications occurred in 15.7 % of cases (n = 39), primarily minor bleeding along the puncture canal (n = 29). Severe complications, including intracerebral hemorrhages (n = 3) and intraventricular hemorrhages (n = 2), were rare, with no other complication occurring more than once. The overall complication rate did not differ significantly between groups: 18.6 % in the EM group (16/86) compared to 14.1 % in the FH group (23/163) (p = 0.35).

### 3.3 Placement characteristics

EM-guided placements were associated with slimmer frontal horns (mean EM: 36.3 mm vs. mean FH: 39.7 mm, p = 0.002), resulting in a significantly lower Evans Index in the EM group (mean EM: 0.28 vs. mean FH: 0.31, p < 0.001) (Table 3). Additionally, EM-guided EVDs were more commonly placed in patients with midline shift (41.9 % vs. 22.7 %, p = 0.002) and on the left side (38.4 % vs. 24.5 %, p = 0.02). Although not statistically significant, first-pass success was slightly higher in the EM group (90.7 %) compared to the FH group (86.5 %).

**Table 3:** Placement characteristics.

Characteristic	Overall N = 249^a^	EMN = 86^a^	FHN = 163^a^	p-value
Title of surgeon				**<0.001**^b^
Introductory position	100 (40.2 %)	45 (52.3 %)	55 (33.7 %)	
Resident	65 (26.1 %)	8 (9.30 %)	57 (35.0 %)	
Consultant	69 (27.7 %)	30 (34.9 %)	39 (23.9 %)	
Senior consultant	15 (6.02 %)	3 (3.49 %)	12 (7.36 %)	
Placement attempts				0.3^b^
1	219 (88.0 %)	78 (90.7 %)	141 (86.5 %)	
2 or more	30 (12.0 %)	8 (9.30 %)	22 (13.5 %)	
Side of placement				**0.023**^b^
Right	176 (70.7 %)	53 (61.6 %)	123 (75.5 %)	
Left	73 (29.3 %)	33 (38.4 %)	40 (24.5 %)	
Midline shift	73 (29.3 %)	36 (41.9 %)	37 (22.7 %)	**0.002**^2^
Midline shift in mm				0.6^c^
Mean, (SD)	5.5, (3.6)	5.4, (3.9)	5.7, (3.4)	
Frontal horn width				**0.002**^d^
Mean, (SD)	38.5, (8.0)	36.3, (8.4)	39.7, (7.5)	
Evans Index				**<0.001**^d^
Mean, (SD)	0.30, (0.1)	0.28, (0.1)	0.31, (0.1)	

Surgical indication				**<0.001**^b^
Infarction	11 (4.42 %)	4 (4.65 %)	7 (4.29 %)	
Infection	27 (10.8 %)	14 (16.3 %)	13 (7.98 %)	
ICH^e^	53 (21.3 %)	17 (19.8 %)	36 (22.1 %)	
IVH^f^ +/− ICH	11 (4.42 %)	1 (1.16 %)	10 (6.13 %)	
SAH^g^	97 (39.0 %)	24 (27.9 %)	73 (44.8 %)	
TBI^h^	32 (12.9 %)	20 (23.3 %)	12 (7.36 %)	
Tumor	16 (6.43 %)	5 (5.81 %)	11 (6.75 %)	
Hydrocephalus UNS	2 (0.80 %)	1 (1.16 %)	1 (0.61 %)	

a n (%).

b Pearson's Chi-squared.

c Wilcoxon rank sum test.

d Welch Two Sample *t*-test EM: Electromagnetic-navigation, FH: Freehand.

e Intracerebral hemorrhage.

f Intraventricular hemorrhage.

g Subarachnoid hemorrhage.

h Traumatic Brain Injury.

The use of EM navigation was significantly correlated with the surgeon's level of experience. Residents, who placed 26.1 % of all external drains, accounted for only 9.3 % of EM-guided placements. Similarly, senior consultants performed three times as many FH EVDs as EM-guided EVDs (12 vs. 4). Conversely, colleagues in an introductory position accounted for 40.2 % of all EVD placements but 52.3 % of EM-guided placements. A similar overrepresentation was observed among consultants, who performed 27.7 % of all EVDs and 34.9 % of EM-guided placements (Table 3). Furthermore, significant differences in surgical indications were noted between the EM-guided and freehand groups (p < 0.001), with EM-guided placement being more commonly used in traumatic cases and less frequently in SAH cases.

## 4 Discussion

In this single-center retrospective study of all EVDs placed over a 21-month period, we reviewed 364 cases and included 249 patients undergoing placement of an external ventricular drain. They were categorized based on placement technique as either freehand (n = 163) or electromagnetically guided (n = 86). We found that the accuracy of EM-guided EVD placement was significantly superior to FH placement, with a 7 % higher accuracy rate based on the Kakarla grading scale. This improved accuracy was achieved despite the EM group facing more anatomically challenging cases, characterized by significantly smaller ventricular systems and a higher frequency of midline shift. Additionally, EM-guided placements were predominantly performed by less experienced surgeons. Despite these challenges, EM-guided EVDs did not exhibit an increased complication rate. Although not statistically significant, EM-guided EVDs also required fewer ventriculostomy attempts, further underscoring the potential advantages of applying this technique.

Previously published rates of FH placement of EVDs report accuracy rates ranging from 66 % to 77 %, as summarized in a recent meta-analysis showing an optimal placement rate of 72 %. (Nawabi et al., 2023), (Stuart et al., 2021) In comparison, the FH accuracy rate in the present study is noticeably higher (84 %), even when compared to previous results from our institution (69.1 %) (Bergdalm, 2013). While this improvement may reflect the continuous advancements in neurosurgical training and techniques, we argue that it is also influenced by the selective application of EM guidance for difficult or complex cases, leaving simpler cases to FH insertions, inflating the accuracy rate.

Furthermore, less experienced physicians, represented in our study as those in an “introductory position,” were disproportionately assigned to the EM group. Given their relative inexperience, it would be expected that they would have a higher misplacement rate. However, we theorize that these surgeons—who benefit the most from additional support—gain significant accuracy improvements from using EM guidance. This dynamic may inadvertently contribute to the higher FH accuracy rate observed in our study.

Our results also show a significantly higher proportion of EM guided EVDs being placed on the left side, in comparison to freehand (Table 2). Although this could be an incidental finding, it could also be another factor, in addition to the higher rate of midline shift and smaller relative ventricular size, indicating more frequent use of EM guidance in complex cases. Traditionally EVDs are initially attempted and placed on the right side, in order to limit potential complications such as aphasia or other speech impairments. This leaves left side ventriculostomies as a last resort, in the case of right-side obstruction, concurrent right sided ventriculostomy or right sided ventricular compression.

Our findings support the routine use of EM guidance in EVD placement, particularly in challenging cases. However, our data do not address potential drawbacks of EM applications, such as the additional training required for both software and hardware proficiency and possibly prolonged operative times. The latter has, however, been reported to differ insignificantly in another EM system (Mahan et al., 2013). Ideally, procedure durations for both EM and freehand (FH) techniques should be compared, but these data were unavailable due to inaccurate or inconsistent recording. Based on the surgeons' experience, use of the Brainlab EM system typically adds approximately 5–10 min to the procedure, depending on the operator's familiarity with the system. Importantly, trajectory planning is performed in the Brainlab software (Elements) on office computers while the patient is being transferred to the OR, minimizing delays. Furthermore, technical challenges are inevitable when introducing new technologies. It remains essential for younger neurosurgeons to master the freehand technique to ensure competence in time-critical situations and when EM guidance is unavailable. Systematically applying EM-guidance when placing EVDs also requires both financial and technical resources not readily available in resource-limited surgical centers.

### 4.1 Limitations

As a single-center retrospective study, our investigation is subject to inherent design constraints. Analyses were not blinded to placement technique, which would have been preferable. Inconsistent procedural records also precluded comparison of EM-guided and free-hand procedure times, leaving any operative-time differences undetermined.

### 4.2 Generalizability

The findings from this study, while promising, must be considered within the context of its single-center design, which may limit the generalizability of the results. The use of EM-guidance demonstrated improved accuracy in ventricular catheter placement despite use in more challenging anatomical scenarios. While our results are encouraging for similar high-volume neurosurgical centers with access to electromagnetic navigation systems, different outcomes might be observed in institutions with less frequent use of such technology or differing patient demographics. Furthermore, the introduction of EM-guidance in neurosurgical training and routine practice may vary significantly based on regional and institutional resources and expertise. Prospective, multicenter studies are needed to validate our findings across broader healthcare settings and to establish standardized protocols that could enhance the reproducibility and applicability of EM-guidance in routine neurosurgical practice.

## 5 Conclusion

This study underscores the efficacy of EM-guidance in enhancing the precision of EVD placements, evidenced by a significant increase in accuracy rates using EM-navigation using fewer passes and without increasing the rate of complications. These outcomes highlight the potential benefits of incorporating electromagnetic navigation into standard neurosurgical practice, particularly for anatomically challenging cases. The consistent accuracy achieved with EM-guidance, despite its use in more anatomically challenging cases with smaller ventricular systems and a higher frequency of midline shifts, supports its wider adoption in neurosurgical practice.

## Author contribution statement

Conceptualization: RR, SHN. Formal Analysis: SHN, KAH. Interpretation of results: All authors. Writing: SHN, KAH, RR. Supervision: RR.

Review and editing of final version: All authors.

## Funding

This study was supported by Brainlab AG, the manufacturer of the electromagnetic navigation system used in this research. Funding was obtained prior to the commencement of the study, and the company had no involvement in study design, data collection, analysis, manuscript preparation, or the decision to submit this work for publication.

## Declaration of competing interest

The authors declare the following financial interests/personal relationships which may be considered as potential competing interests:Silas Haahr Nielsen and Rune Rasmussen reports financial support was provided by Brainlab AG. If there are other authors, they declare that they have no known competing financial interests or personal relationships that could have appeared to influence the work reported in this paper.
